# Therapeutic Drug Monitoring of Posaconazole: an Update

**DOI:** 10.1007/s12281-016-0255-4

**Published:** 2016-05-07

**Authors:** Bart G. J. Dekkers, Martijn Bakker, Kim C. M. van der Elst, Marieke G. G. Sturkenboom, Anette Veringa, Lambert F. R. Span, Jan-Willem C. Alffenaar

**Affiliations:** Department of Clinical Pharmacy and Pharmacology, University of Groningen, University Medical Center Groningen, PO Box 30.001, 9700 RB Groningen, The Netherlands; Department of Hematology, University of Groningen, University Medical Center Groningen, Groningen, The Netherlands; Department of Clinical Pharmacy, ZGT Hospital Group Twente, Hengelo, The Netherlands

**Keywords:** Posaconazole, Therapeutic drug monitoring, Pharmacokinetics, Pharmacodynamics, Invasive fungal infection, Invasive aspergillosis, Prophylaxis, Dried blood spot, Mucormycosis, *Scedosporium* infections, *Fusarium* infections

## Abstract

Posaconazole is a second-generation triazole agent with a potent and broad antifungal activity. In addition to the oral suspension, a delayed-release tablet and intravenous formulation with improved pharmacokinetic properties have been introduced recently. Due to the large interindividual and intraindividual variation in bioavailability and drug-drug interactions, therapeutic drug monitoring (TDM) is advised to ensure adequate exposure and improve clinical response for posaconazole. Here, we highlight and discuss the most recent findings on pharmacokinetics and pharmacodynamics of posaconazole in the setting of prophylaxis and treatment of fungal infections and refer to the challenges associated with TDM of posaconazole.

## Introduction

Invasive fungal disease (IFD) is associated with substantial morbidity and mortality [1]. Infections with *Candida* spp. are most often observed in hematology-oncology and surgical patients [1]. Invasive aspergillosis (IA) occurs most frequently in neutropenic hematology-oncology patients and solid organ transplant and hematopoietic stem cell transplant patients [1]. Among solid organ transplant patients, lung transplant recipients are particularly at risk for IA [1].

Posaconazole is a second-generation triazole agent with a potent and broad antifungal in vitro activity against a range of different fungal pathogens, including *Aspergillus* spp. and *Candida* spp. It is structurally related to itraconazole and inhibits lanosterol 14α-demethylase (CYP51), blocking the synthesis of ergosterol resulting in impaired cell membrane stability and accumulation of precursors leading to fungistatic or fungicidal effects [2]. Besides facing the increasing prevalence of resistant fungi [3•, 4], personalized treatment to increase efficacy and avoid toxicity is urgently needed. Therapeutic drug monitoring (TDM), in combination with clinical assessment of response and determination of minimum fungicidal inhibitory concentration (MIC), may help to optimize treatment results. In this review, we present recent findings on pharmacokinetics and pharmacodynamics of posaconazole in the setting of prophylaxis and treatment of fungal infections. In addition, we evaluate the effects of these recent findings on TDM of posaconazole in daily practice. For selected topics, we refer to earlier published reviews for in-depth discussion.

### Pharmacokinetics of (New) Posaconazole Drug Formulations

Posaconazole is currently available in three formulations. The oral suspension was introduced in 2005. The pharmacokinetic profile of the suspension has been outlined extensively [5, 6]. In short, the use of the posaconazole suspension has been limited due to satiable absorption and variable bioavailability necessitating the administration in divided doses three to four times per day. The bioavailability is also strongly dependent on the concomitant intake of food, gut motility, and gastric acidity. The absorption of posaconazole is significantly increased when administered with a (high-fat) meal [7]. Several studies have shown subtherapeutic posaconazole concentrations in patients with no or limited food intake or after administration by nasogastric tube [8–10]. Furthermore, co-administration of the suspension with a proton pump inhibitor, H_2_ antagonist, or metoclopramide resulted in subtherapeutic posaconazole exposures, due to a reduced absorption secondary to a decrease in gastric acid production or increased gut motility [8–12]. The suspension is therefore not preferable for patients who use these drugs or are unable to eat. Furthermore, besides these exceptions, it is difficult to obtain consistent therapeutic levels in general practice, necessitating frequent TDM [9•, 13].

Recently, a delayed-release tablet and intravenous formulation have been introduced [14, 15•]. The delayed-release, gastro-resistant film-coated tablet consists of a pH-sensitive polymer stabilizer excipient that limits posaconazole release at low pH in the stomach and releases posaconazole at neutral pH in the small intestine [16]. Compared to the suspension, the tablet shows an improved bioavailability in healthy subjects, resulting in an approximately fourfold increase in maximum concentration (*C*_max_) and a threefold increase in the exposure (expressed as area under the concentration-time curve (AUC)) under fasted conditions [17]. In contrast to the suspension, the posaconazole exposure after administration of the tablet is only moderately affected by food. The posaconazole exposure increased by 1.5-fold when the tablet was administered with a high-fat meal than when administered in the fasted state, compared to a fourfold increase in exposure with the suspension [18]. Under fed conditions, exposure was approximately 35 % higher for the tablet formulation compared to the suspension [17]. Importantly, exposure for the tablet does not appear to be markedly affected by drugs that influence gastric acidity or gut motility [14, 17]. Other advantages of the tablet formulation are the once daily administration and reduced interpatient variability [19]. Moreover, the tablet showed linear pharmacokinetics for the tested dosage range (up to 400 mg) [20]. A limited role may remain for the oral suspension, for example, in the treatment of patients who are unable to take tablets, such as patients with dysphagia, children, or patients with enteral feeding tubes [8], as the tablet must be swallowed whole (not divided, crushed, or chewed). For these patients, TDM should be applied to assure adequate exposure (see also below).

The intravenous formulation has also the advantage of a once daily administration and is suitable for patients who cannot tolerate oral medication [15•, 19, 21]. A disadvantage of the intravenous formulation is the need to administrate via a central venous catheter and the presence of the solubilizing excipient sulfobutylether-β-cyclodextrin, which may accumulate in patients with moderate to severe renal impairment potentially leading to (additional) renal toxicity [22]. Although the volume of distribution appeared to be increased for the suspension, no clinically relevant differences were observed between the other pharmacokinetic parameters of the suspension, the tablet, and the intravenous formulation (Table 1) [23].

**Table 1:** Pharmacokinetic parameters for posaconazole oral suspension, delayed-release tablet, and IV solution in healthy volunteers

	Formulation		
	Oral suspension	Delayed-release tablet	IV solution
Recommended dose			
- Refractory IFD/intolerance to first-line therapy	200 mg four times a day, 400 mg twice daily in combination with food	Loading dose 300 mg twice daily on day 1 followed by 300 mg once daily	Loading dose 300 mg twice daily on day 1 followed by 300 mg once daily
- Oropharyngeal candiasis	200 mg loading dose on day 1, then 100 mg once daily for 13 days in combination with food	N/A	N/A
- Prophylaxis of IFD	200 mg thrice daily in combination with food	Loading dose 300 mg twice daily of day 1 followed by 300 mg once daily	Loading dose 300 mg twice daily of day 1 followed by 300 mg once daily
*V*_*D*_	1774 L	394 (294–583) L	261 L
*T*_max_	3 h	4–5 h	90 min (end of infusion)
Protein binding	98 %	98 %	98 %
*t*_1/2_	35 (20–66) h	29 (26–31) h	27 h
Elimination (percent of radiolabelled dose)	Feces (77 %)	Feces (77 %)	Feces (77 %)
	Renal (14 %)	Renal (14 %)	Renal (14 %)
Time to reach steady state	7–10 days	6 days	6 days
Food-drug interaction	Increased *C*_max_ (330 %) and AUC (360 %) in combination with a high-fat meal	Increased *C*_max_ (51 %) and AUC (16 %) in combination with a high-fat meal	N/A
Drug-drug interactions	Drugs affecting gut motility, gastric pH, and P-gp enzyme-inducing drugs. Posaconazole inhibits CYP3A4	P-gp enzyme-inducing drugs. Posaconazole inhibits CYP3A4	P-gp enzyme-inducing drugs. Posaconazole inhibits CYP3A4

*Source*: [23]

*IFD* invasive fungal disease, *V*_*D*_ volume of distribution, *T*_max_ time until the maximum serum concentration, *t*_*1/2*_ half-life, *N/A* not applicable

With the introduction of the new posaconazole formulations, two additional suitable treatment options can be chosen, which have significantly improved the pharmacokinetics and clinical utility of this antifungal agent compared to the oral suspension [24].

### Pharmacokinetic/Pharmacodynamic Relationships for Posaconazole

Pharmacokinetics describe the behavior of a drug in a patient’s body, including absorption, distribution, metabolism, and excretion, whereas pharmacodynamics describe the biochemical or pharmacological effect of the drug on the patient’s body or, in case of infectious diseases, the pathogen [25]. Together, both parameters represent the time-effect course of a drug after administration in relation to the biochemical or pharmacological effect. For antifungal drugs, the pharmacodynamics are related to the MIC. Studies in in vitro and in vivo models indicate that the ratio of the total posaconazole AUC over 24 h over the MIC best represents the pharmacokinetic/pharmacodynamic index for posaconazole in the treatment of IA [3•]. AUC/MIC ratios of 167 to 187 were found to be predictive of successful treatment of *Aspergillus* spp. [3•]. Similarly, also for *Rhizopus oryzea* mucormycosis, an AUC/MIC of >100 has been shown to be sufficient [26]. In practice, an AUC/MIC ratio of 200 is advised for infections with *Aspergillus* spp., corresponding to a *C*_min_/MIC ratio of 5-8 (Table 2) [23]. For prophylaxis, a total posaconazole AUC/MIC ratio of at least 94 was recently found to be predictive of success [27].

**Table 2:**
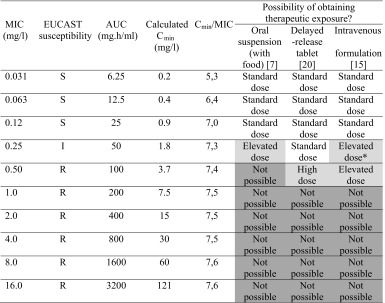
Possibility of obtaining a therapeutic exposure in the treatment of invasive aspergillosis for the different posaconazole formulations. Data represent values calculated for an AUC/MIC ratio of 200. Due to the linear pharmacokinetics, values can be divided by two for an AUC/MIC ratio of 100 in the prophylaxis setting. Table adapted from [3•]

*MIC* minimum inhibitory concentration, *EUCAST* EUropean Committee on Antifungal Susceptibility Testing, *AUC* area under the concentration time curve, *C*_*min*_ trough concentration, *S* susceptible, *I* intermediate, *R* resistant

^a^For MIC values of 0.25 mg/l, AUC/MIC ratios cannot be reached with the standard dose as the registered dose for intravenous use is 300 mg, whereas the registered dose for the tablets is 400 mg [23]

**Fig. 1:**
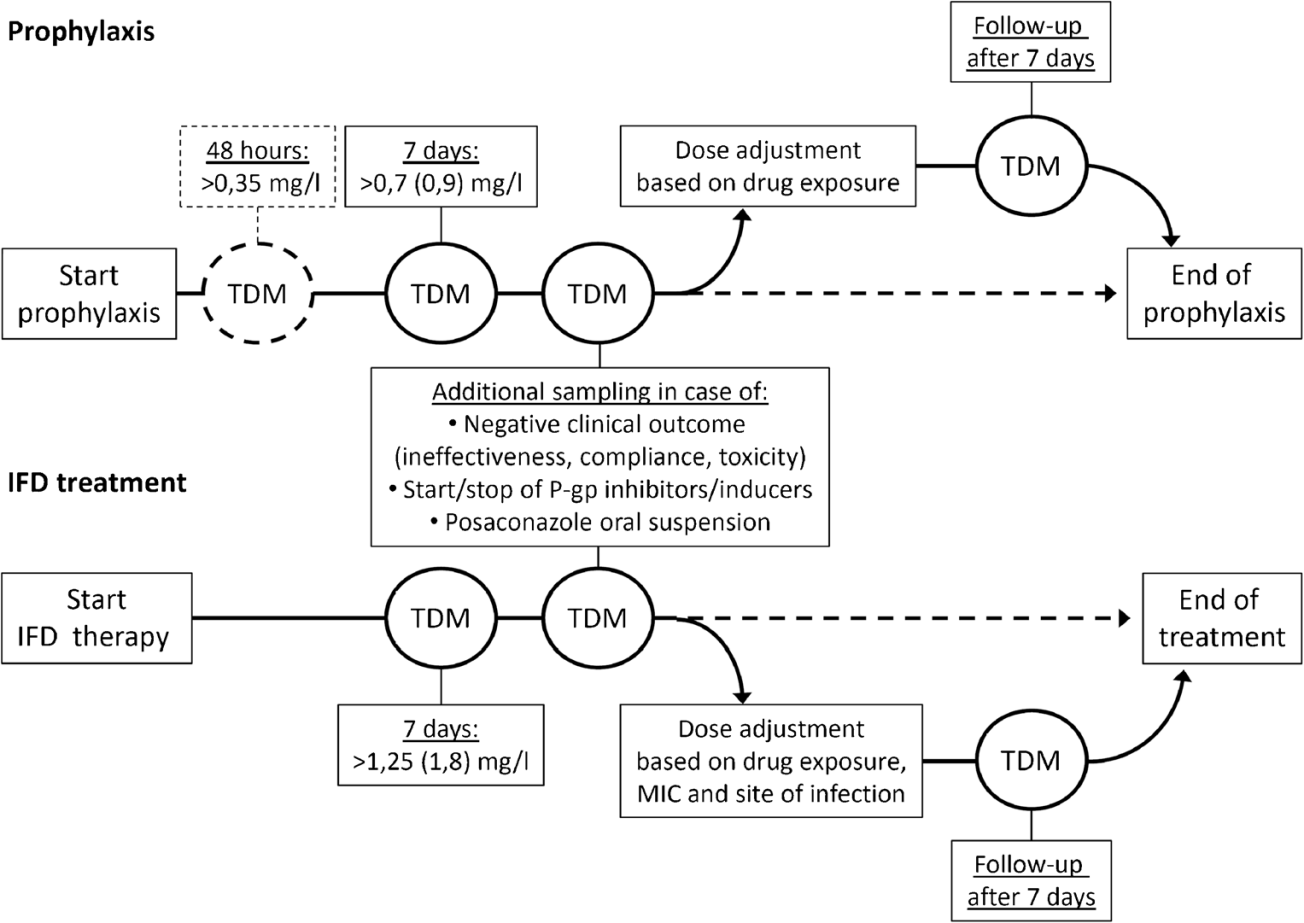
Therapeutic drug monitoring (TDM) of posaconazole. TDM is recommended after 7 days of treatment for posaconazole in case of the salvage treatment of invasive fungal infections, interacting drugs (P-gp inhibitors), of use of the posaconazole oral suspension and in case of specific clinical circumstances. In case of salvage treatment, TDM is also required when a pathogen with reduced susceptibility (>0.12 mg/l) to posaconazole is isolated or when the pathogen is localized at a difficult to reach site. If the trough level is above 0.7 (0.9) mg/l (AUC/MIC > 100) for prophylaxis or above 1.25 (1.8) mg/l (AUC/MIC > 200) for salvage treatment, the dose should be maintained. In case of a trough level below these target concentrations, effort should be done to increase the concentration to target levels. *IFD* invasive fungal disease, *MIC* minimum fungicidal inhibitory concentration, and *P-gp* P-glycoprotein

Due to the large interindividual and intraindividual variations in bioavailability and drug-drug interactions, TDM has been proposed as a tool to ensure adequate exposure and improve clinical response for posaconazole [9•, 28–30•]. Although much debated, clinical studies suggest trough levels of >0.7 mg/l for prophylaxis and trough levels of >1.0–1.25 mg/l for treatment of IFD (Fig. 1) [13, 29]. However, these concentrations were established independently of the susceptibility of the invading fungal pathogen. For the suspension, the proposed targets could only be reached by a limited number of subjects for less sensitive strains of *Aspergillus* spp., which are still considered to be susceptible (MIC ≤ 0.12 mg/l; Table 2) [3•, 31]. With the introduction of the tablet and intravenous formulations, these targets can be reached more easily, making it also possible to even treat subjects with an intermediate sensitive *Aspergillus* strains, although high doses are likely to be required. In clinical practice, the tablet formulation is preferred over the oral suspension as median posaconazole plasma concentrations increased from 0.75 to 1.9 mg/l in leukemia patients after switching from the suspension to the solid formulation. Fortunately, study participants experienced no additional toxicity after the switch [32]. Exposure may, however, remain also an issue for the tablets under more extreme conditions as lower posaconazole trough levels were observed in patients weighing ≥90 kg or in patients with a body mass index ≥30 [33]. Patients with diarrhoea also showed lower trough levels on the tablets [33]. For this group and other patients with absorption problems, the intravenous formulation may be a valuable addition.

Posaconazole accumulates in lung, kidney, heart, and liver tissue, but not in the brain. Brain and plasma concentrations were approximately equal, suggesting that higher plasma concentrations may be required for brain infections [34]. Levels in cerebrospinal fluid were found to be variable, suggesting that diffusion of posaconazole into the brain is increased with meningeal inflammation [35]. Moreover, TDM of posaconazole may become increasingly important to ensure adequate exposure and thereby prevent the emergence of posaconazole-resistant strains [36]. Currently, no concentration-dependent adverse events or toxicity have been described for posaconazole [29], although an upper boundary of 3.75 mg/l is suggested for the average posaconazole plasma concentrations by the European Medicines Agency [16]. With the introduction of the new dosage forms, these higher levels may be reached which could result in toxicity for which TDM may become relevant as well. Recently, it was shown that 3 % of patients treated with the tablets have trough levels of ≥3.75 mg/l [37•]. In line with this assumption, increasing the dose of the intravenous formulation from 200 to 300 mg resulted in an increase in adverse events (diarrhea, mucosal inflammation, headache, and rash) [15•]. Moreover, use of the new tablet and intravenous formulations may result in new adverse events as was already observed for the intravenous formation, which showed a high number of infusion reactions after peripheral administration [21]. Collectively, these findings indicate an essential role for TDM in the treatment of invasive fungal infections with posaconazole. TDM can be further refined with MIC measurements. For empirical treatment and pre-emptive of IFD, we suggest target trough levels of 1.8 mg/l to cover all sensitive strains. For prophylaxis, a target trough level of 0.9 mg/l can be suggested based on AUC/MIC ratios (Table 2), although a trough level of >0.5 mg/l was recently found to be effective in hematology patients treated with posaconazole tablets [37•]. For the new tablet and intravenous formulations, TDM should be applied in particular in the treatment of IFD as trough levels of ≥1.8 mg/l are expected not be reached in approximately 20 % of patients [38]. For use of these formulations in prophylaxis, TDM should only be performed in exceptional cases as target levels are expected to be reached in almost all the patients (Fig. 1) [26]. Use of the posaconazole suspension should be restricted to a minimum and should always be associated with TDM. Clinical studies are warranted and currently ongoing to establish and increase the level of evidence for the use of TDM, especially for the tablet and intravenous formulations [39 •].

### Alternative Sampling Procedures for TDM

In hospitalized patients, TDM of posaconazole can easily be performed in serum or plasma. For outpatient monitoring, sampling may be more problematic. Alternatively, sampling for posaconazole has been performed using the new dried blood spot (DBS) technique [40•, 41]. Studies are ongoing to validate this sampling method in children [42]. With DBS sampling, blood is obtained using a finger prick instead of a venous blood sample. After receiving an instruction in DBS sampling, patients can obtain the DBS samples themselves at home and sent them by mail to the laboratory for analysis. Besides the less invasive sampling procedure, DBS analysis has the advantage of a smaller sampling volume, simpler storage, and transfer of samples at room temperature, without biohazard risks during the shipment. One study evaluated the patients’ opinions of the sampling method and showed that patients were satisfied with DBS sampling, and most patients preferred DBS over venous blood sampling [40 •]. With DBS analysis, the possibilities of TDM for posaconazole can be extended to patients at home and to hospitals without a bioanalytical infrastructure [40 •, 43].

### TDM of Posaconazole in the Treatment of IA

Although posaconazole is similar to voriconazole in its activity against *Aspergillus* species, use of posaconazole is preserved for salvage therapy in patients who are refractory or intolerant to voriconazole [36, 44, 45]. In case of voriconazole treatment failure, a switch of antifungal drug class is generally recommended [44]. Nevertheless, compared with amphotericin B, itraconazole, voriconazole, or echinocandins, posaconazole is associated with higher response rates [46]. The efficacy and safety of the posaconazole oral suspension, which was the only available formulation at that moment, as monotherapy was investigated in patients with IA who were refractory or intolerant to conventional antifungal therapy and was found both save and effective (40–70 % of patients) as salvage treatment for patients which had previously been treated with another triazole [44, 46]. Given these results and its spectrum of activity, posaconazole may be an effective primary agent for the treatment of IA as well [45]. However, the place of posaconazole as first-line treatment should be tested in a randomized, controlled trial comparing the intravenous and tablet formulations against the current standard therapy (voriconazole with TDM), before recommendation as initial therapy [45, 46]. This study is currently ongoing [47].

Azole resistance is an emerging problem for *Aspergillus* species [3•, 4]. The majority of reports concern *Aspergillus fumigatus*, although azole resistance has been reported sporadically in other species as well [3•]. Azole-resistant *A. fumigatus* isolates have been reported in several countries around the world, and clinical failures have been attributed to microbiological resistance [4]. A wide range of mutations in *A. fumigatus* have been described conferring azole resistance commonly involving modifications in the CYP51 gene [3•, 4, 48], the target of antifungal azoles. Acquired resistance may be developed in patients with chronic cavitating aspergillosis treated after long-term azole exposure, when a susceptible isolate obtains the ability to resist the activity of the antifungal agent [3•, 36, 49]. In addition, increasing agricultural use of azole compounds over many years is held responsible for the environmental contamination (acquired resistance), leading to primary resistant isolates in azole-naive patients [3•, 36]. As long-term therapy of aspergillosis is required in most individuals, and the azoles are the only clinically available agents that can be administered orally, the development of azole resistance in *A. fumigatus* is worrisome [4]. If a role for the azoles remains in the management of azole-resistant aspergillosis, optimizing drug exposure is critical to increase the probability of treatment success [3•]. In this context, measuring MIC values to the azole compounds is crucial to increase the clinical response [3•]. Seyedmousavi et al. proposed break points of 0.25 to 0.5 mg/l for posaconazole, which are higher than the EUCAST break points for *Aspergillus* spp. [3•, 31, 50]. Reduced susceptibility to azoles has significant impact on the ability to achieve the pharmacodynamic target, and sometimes, targets can only be achieved at the cost of increased toxicity. Posaconazole exposure (estimated by the AUC) correlates linearly with the dose; thus, a higher dose of the azole is required to achieve similar efficacy when azole-resistant strains are present (Table 2) [3•]. With the conventional suspension and dosing of 200 mg four times a day, sufficient exposures may be difficult to attain. However, such levels may be obtained with the new posaconazole delayed-release tablets (Table 2). A case report of a patient with a cerebral IA which was successfully treated with the tablet formulation has been recently published [51]. The patient has been treated for a brain abscess with voriconazole for 1 year, but MRI imaging showed a new frontal epidural fluid collection. It was assumed that susceptibility to voriconazole was reduced, and therefore, the patient was switched to posaconazole tablets, 300 mg twice daily. The trough level after 2 weeks of treatment was 5.3 mg/l. The dose was reduced to 300 mg once daily, leading to a trough level of 2.0 mg/l [51]. As cultures were negative, they were unable to determine MICs. The patient responded well to the posaconazole, and a repeated MRI of the brain 4 months after posaconazole initiation showed a significant improvement consistent with the resolving infection [51].

Clinical effectiveness of posaconazole salvage treatment has been shown to be dependent on posaconazole plasma levels in an externally controlled study [13]. Higher plasma concentrations of posaconazole were associated with greater response rates. For patients with average plasma concentrations of >1.25 mg/l, clinical effectiveness was increased to 75 % compared to 24–53 % for patients with lower plasma levels [13]. Importantly, these levels were only reached in 24 % of the patients in this study with the oral suspension [13]. Moreover, based on the AUC/MIC targets indicated in Table 2, target trough levels of 1.8 mg/l are suggested, which can be reached with the new formulations in most but not all patients, and therefore, TDM is warranted to assure efficacy (Fig. 1) [30 •]. Unlike the prophylaxis setting [52], no early target (48 h) levels are currently available to assure adequate exposure early in treatment.

### TDM of Posaconazole in the Setting of Prophylaxis

IFD is associated with a high mortality and is difficult to treat [1]. Preventing these infections could possibly increase survival of immunocompromised patients at risk, including patients with acute leukemia who are especially vulnerable because of the long period of neutropenia during treatment with chemotherapy. Antifungal prophylaxis with fluconazole has been standard of care for patients undergoing intensive remission-induction chemotherapy or hematopoietic stem cell transplantation for the last two decades [53]. However, fluconazole lacks activity against invasive mold infections, thereby limiting its possibilities in preventing these infections. Contrary to this, posaconazole has an extended spectrum of activity, including filamentous fungi like *Aspergillus*, *Zygomycetes*, and *Fusarium* species.

Two landmark clinical trials support the use of posaconazole as prophylaxis against IFD. A randomized multi-center open-label study by Cornely et al. found that posaconazole prophylaxis was associated with a significant reduction in IFD and improved overall survival (16 versus 22 %) compared to itraconazole and fluconazole prophylaxis in patients with neutropenia secondary to chemotherapy for acute myelogenous leukemia or the myelodysplastic syndrome [54]. Another trial by Ullmann et al. found that posaconazole was as effective as fluconazole in preventing IFD. Posaconazole did show significant superiority in preventing probable or proven aspergillosis. Overall mortality was similar between the two treatment groups, but death due to IFD was lower in the posaconazole group (1 versus 4 %) [55]. Both studies were performed with posaconazole oral suspension. After the publication of the multi-center trials, several real-life experiences confirmed the efficacy of posaconazole prophylaxis with the suspension in the clinical setting [56–62]. These observational studies have several limitations; they were partly retrospective and some had historical controls, but all, except one small study [62], showed a significant reduction in IFD [56–61, 63, 64]. Most studies did not show a reduction in all-cause mortality, except two [58, 61]. The combined level of evidence is reason for all international guidelines to recommend the use of posaconazole as antifungal prophylaxis in the hematopoietic stem cell transplantation recipients with GvHD and in neutropenic patients with acute myelogenous leukemia or myelodysplastic syndrome who are at high risk for IA [65].

Target trough levels of 0.7 mg/l after 7 days of treatment are currently being advised by guidelines for TDM of posaconazole prophylaxis. The rationale for this through level is based on an analysis by the FDA on the pharmacokinetic data from the studies by Ullmann et al. and Cornely et al. [37•, 54, 55, 66]. Clinical failure was 25 % at this posaconazole plasma level and did not improve much at higher concentrations [66]. This relatively high number of events could be due to a number of aspects, including variation in tissue concentrations and variation in exposure due to use of the oral suspension [26, 34]. In addition to the target level of 0.7 mg/l, a trough level of >0.35 mg/l after 2 days of treatment has been suggested as predictive for an appropriate trough level after 1 week of treatment with the oral suspension [52]. Approximately 50 % of the patients had serum levels below the threshold of <0.7 mg/l when using the posaconazole suspension [30•]. For the tablets, 90 % of patients had trough levels ≥0.7 mg/l and only 5 % had levels <0.5 mg/l [37•]. In addition, several smaller and retrospective studies have shown a positive correlation between the posaconazole exposure and therapeutic efficacy [30•], supporting the use of TDM when using the oral suspension. Moreover, based on the AUC/MIC targets indicated in Table 2, we recommend target trough levels of 0.9 mg/l, which can be reached with the tablet formulation in the majority of patients [26]. In patients treated with posaconazole for prophylaxis, TDM seems to be of particular relevance in case of drug-drug interactions, toxicity, or use of the oral suspension (Fig. 1).

### Treatment of Other Fungi with Posaconazole

Although *Aspergillus* infections are commonly seen in immunocompromised patients, other opportunistic fungal infections, like mucormycosis, may also occur. Mucormycosis is a rare fungal infection with a high mortality. Moreover, the prevalence of this life-threatening fungal infection is increasing, partly because of increasing resistance to voriconazole [67]. In contrast to the other triazoles, most *Mucorales* infections are susceptible to posaconazole. The first-line treatment of mucormycosis is liposomal amphotericin B, which shows a good efficacy for the majority of the strains. Posaconazole is recommended as salvage therapy, and most strains are susceptible for posaconazole, except for *Mucor circinelloides*. The survival rates in patients who used posaconazole as salvage therapy are described in two series and were found to be 62 and 79 % [67, 68]. In one study, random serum posaconazole trough levels were assayed; however, no relationship with clinical efficacy was provided [68]. There is limited data on survival rate and the susceptibility of posaconazole as first-line treatment for mucormycosis. In addition to antifungal treatment, surgery is highly recommended [67, 69, 70]. The main reasons for switching to posaconazole in clinical practice are treatment failure or toxicity, especially nephrotoxicity with long-term first-line treatment with amphotericin B, or the need for oral treatment as a step down for successfully treated patients [71]. The new formulations of posaconazole have not been studied in patients with mucormycosis; however, they have been successfully applied in neutropenic murine models. Based on the findings in these models, a Monte Carlo simulation was performed, which showed that with the new formulations of posaconazole, the target AUC/MIC was achieved in almost all the simulated patients (95–97 %) for an MIC up to 0.12 mg/l for *Rhizopus oryzae*, while with the suspension, the AUC/MIC target was only achieved for an MIC up to 0.03 mg/l in almost all simulated patients (96–97 %) [26]. Therefore, the new formulation of posaconazole can be a very promising alternative treatment of mucormycosis. Pharmacodynamics of posaconazole for *R. oryzea* were comparable to *A. fumigatus*. Therefore, similar target trough levels should be pursued, taking the MIC into account (Table 2) [26].

Furthermore, posaconazole exhibits relatively consistent activity against *Scedosporium* spp., as well as voriconazole, although in vitro susceptibility to voriconazole and posaconazole is highly variable. For instance, *Scedosporium prolificans* seems to be resistant for both voriconazole and posaconazole. Therefore, antifungal susceptibility testing plays a crucial role in the treatment of *Scedosporium* infections. On average, the MIC values for voriconazole are lower than those for posaconazole [69, 72, 73]. However, the pharmacokinetics of voriconazole are highly variable in clinical practice, which results in variable plasma concentrations of voriconazole as well [74]. In addition, more adverse effects are observed for voriconazole compared to posaconazole. Therefore, the posaconazole tablets can have an advantage over voriconazole for the treatment of posaconazole susceptible *Scedosporium* infections. Other triazoles and polyenes, including amphotericin B deoxycholate and lipid amphotericin B formulations, have no or limited activity against these pathogens [69, 72]. Other infections that are commonly seen in immunocompromised patients are infections caused by *Fusarium* spp. The optimal treatment strategy is not yet fully established for these infections, because clinical trials are lacking. Voriconazole is considered first-line treatment in immunocompromised patients with *Fusarium* infections. Posaconazole can be used as salvage therapy. Other older azoles show reduced activity against these organisms [73, 75]. The susceptibility against *Fusarium* spp. is also highly variable for voriconazole and posaconazole. In addition, in vitro susceptibility testing showed that amphotericin B was the most potent antifungal agent. The meaning of these in vitro findings for clinical practice are unknown. Therefore, as for *Scedosporium* infections, posaconazole should only be prescribed once susceptibility testing has been performed and susceptibility of the *Fusarium* strain has been shown [76]. It is not clear whether for other species similar pharmacokinetic/pharmacodynamic targets should be achieved as currently determined for *A. fumigatus* and *R. oryzae*. Future studies are therefore warranted. Unfortunately, no studies have been performed or are ongoing investigating the role of TDM in these infections. But in general, it is accepted that higher posaconazole exposure is needed than required for treatment of IA and that TDM should be used to optimize treatment of other fungal infections as well [69].

### Challenges in Posaconazole TDM

Posaconazole has gained a solid position in prophylaxis and salvage therapy of IFD [77]. Due to the availability of the new formulations [14, 15•], posaconazole may increasingly being used as treatment in situations in which voriconazole may not be adequate due to possible resistance, drug-drug interactions, or intolerance. Currently, a phase III study is ongoing comparing posaconazole with voriconazole with TDM as first-line treatment for IA [47]. More information on TDM of posaconazole is urgently needed. To date, only retrospective or prospective observational data on the association between the posaconazole plasma concentration and efficacy is available [46, 54, 55, 66]. Data from a prospective randomized controlled trial comparing posaconazole with and without TDM is currently lacking. Such a trial would be needed to add TDM of posaconazole to standard care in the setting of both prophylaxis and treatment. However, a randomized TDM trial is not likely to be performed in the setting of prophylaxis due to the large sample size and study costs. In addition, in the setting of salvage treatment, patients’ conditions and infections are very heterogeneous, making a design with two comparable arms complicated. More importantly, it may no longer be ethical to withhold TDM in a salvage setting as this is standard practice as shown by the participating of a wide range of laboratories in a proficiency testing program for antifungal drugs [78]. On the other hand, do we actually need level A grade evidence to perform TDM? If we consider TDM a simple tool to assess drug exposure and accept a level B/C grade of evidence, we could use TDM of posaconazole in a targeted population of patients experiencing adverse drug reactions or showing no clinical response to treatment. Compared to other diagnostic procedures, like imaging with PET-CT or biomarker monitoring (galactomannan), measuring a drug level is relatively cheap. As antifungal treatment is very expensive, preventing escalation to combination treatment by determining a blood concentration, TDM can easily be cost-effective.

These challenges should actually not withhold us from collecting the evidence to support TDM. Only the classical randomized control trial approach may not be the best strategy to collect the evidence. Innovative trial designs in a setting of frequent fungal infections may help to collect the evidence for TDM in a salvage setting. For example, a multi-arm multi-stage (MAMS) trial design that proved its use in similar complex infectious diseases, like tuberculosis [79], may be useful for the evaluation of antifungal treatment guided by TDM. During interim analyses, the arms performing less than control are dropped and recruitment for that arm is stopped. Finally, the remaining novel strategy is compared to standard treatment on a relevant clinical endpoint.

## Conclusion

Due to the new formulations, the role of posaconazole in the treatment of fungal infections is likely to increase. TDM of posaconazole is therefore likely to expand as well (Fig. 1). TDM is currently supported by limited results from several prospective cohort studies. In a salvage setting, TDM of posaconazole can be considered as standard of care. To increase the level of evidence to support TDM in other situations, innovative trial designs have to be employed to make prospective randomized controlled studies feasible. Moreover, studies are warranted on early (12–48 h) target levels for the new dosage forms to assure adequate posaconazole exposure in fragile patients.
